# Seromucinous borderline ovarian tumors: Clinical and ultrasound characteristics and association with endometriosis

**DOI:** 10.1111/aogs.70254

**Published:** 2026-05-26

**Authors:** Simona Del Forno, Marisol Doglioli, Chiara Landolfo, Giovanna Palomba, Claudia Vicenzi, Matilde Antonelli, Lucia De Meis, Francesca Govoni, Roberto Paradisi, Diego Raimondo, Renato Seracchioli

**Affiliations:** ^1^ Division of Gynaecology and Human Reproduction Physiopathology IRCCS Azienda Ospedaliero‐Universitaria di Bologna Bologna Italy; ^2^ Department of Medical and Surgical Sciences, DIMEC University of Bologna Bologna Italy; ^3^ Queen Charlotte's and Chelsea Hospital Imperial College Healthcare NHS Trust London UK; ^4^ Department of Metabolism, Digestion & Reproduction, Faculty of Medicine Imperial College London London UK

**Keywords:** borderline ovarian tumors, endometriosis, ovarian cyst, ovary, sonography

## Abstract

**Introduction:**

Seromucinous borderline ovarian tumors represent a distinct borderline ovarian tumor histotype, yet their specific sonographic features and clinical associations remain undercharacterized. The objective of this study was to describe the ultrasound and clinical characteristics of seromucinous borderline ovarian tumors compared to other borderline tumor histotypes and to evaluate their association with endometriosis.

**Material and Methods:**

This single‐center observational retrospective cohort study was conducted at a tertiary‐level reference ultrasound unit. The primary outcome was to analyze the clinical and ultrasound characteristics of seromucinous borderline ovarian tumors compared to other histotypes. The secondary outcome was to evaluate their association with different endometriotic lesions. In women with borderline ovarian tumors, anamnestic data (age, body mass index, parity, menopausal status), preoperative serum carbohydrate antigens 125 and carbohydrate antigen 19.9 levels, ultrasound characteristics (according to the International Ovarian Tumor Analysis group), surgery type, and histological data, including endometriosis localization (superficial, deep, or ovarian), were extracted from medical records.

**Results:**

Among 151 women with borderline ovarian tumors, five had rare histotypes and were excluded. Of the 146 included women, 96 (63.6%) had a serous histotype, 27 (17.9%) a mucinous histotype, and 23 (15.2%) had a seromucinous histotype. Seromucinous borderline ovarian tumors were more commonly left‐sided, unilateral unilocular or multilocular solid cysts, with ground‐glass content, showing moderate vascularization at color Doppler examination (Color Score 3) of papillary projections/solid component. Moreover, they had higher preoperative levels of carbohydrate antigen 19.9 compared to other histotypes (*p* < 0.001). Endometriosis was detected in 26% of all study women but was significantly more frequent (70%) in women with seromucinous borderline ovarian tumors (*p* < 0.001).

**Conclusions:**

Compared to other histotypes, seromucinous borderline ovarian tumors appeared more commonly as left unilateral unilocular or multilocular solid cysts, with ground‐glass content, showing moderate vascularization of papillary projections/solid component. Moreover, they showed a higher increase in serum carbohydrate antigen 19.9 and were associated with endometriosis in most cases.

## Graphical abstract

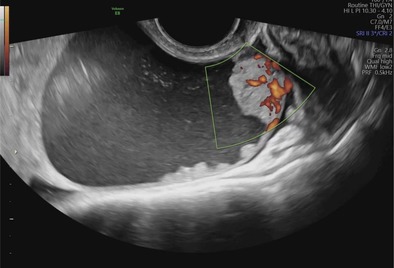

**Abbreviations**
BOT: Borderline ovarian tumor
CA‐19.9: Carbohydrate antigen 19.9
CI: Confidence interval
CS: Color score
MBOT: Mucinous borderline ovarian tumor
OR: Odds ratio
SBOT: Serous borderline ovarian tumor
SMBOT: Seromucinous borderline ovarian tumor
WHO: World Health Organization

**Key message**

Seromucinous borderline ovarian tumors frequently appear as unilateral, left‐sided cysts with ground‐glass echogenicity and moderately vascularized solid components. Their strong association with ipsilateral endometriosis provides crucial sonographic and clinical clues for an accurate preoperative diagnosis.

## 1 INTRODUCTION

Borderline ovarian tumors (BOTs) are defined as a heterogeneous group of epithelial ovarian tumors characterized by atypical cell proliferation and absence of stromal invasion.^1^ BOTs account for 10–15% of all epithelial ovarian tumors and appear to result from failure of cyclic ovulation and aging of ovarian tissue causing metaplasia with subsequent dysplastic transformation when exposed to hormonal and inflammatory stimuli.^2^, ^3^ BOTs are diagnosed in 35% of cases under the age of 40.^4^ They are typically diagnosed at an early stage, with 75% of cases detected at stage I according to the International Federation of Gynecology and Obstetrics (FIGO) staging system, and their prognosis is excellent, with a 10‐year survival rate of 97%.^5^, ^6^

In 2014, the World Health Organization (WHO) revised the histological classification of BOTs, taking into consideration their morphological and biomolecular characteristics (i.e., *KRAS* mutations, *ARID1A* alterations, hormone receptor expression).The new classification included six different classes of BOTs: serous (SBOTs) (55% of cases), mucinous (MBOTs) (35–45% of cases), seromucinous (SMBOTs) (5–7% of cases); Brenner (3–5% of cases), endometrioid (2–3%), and clear cell (<1%).^7^ The novelty of this 2014 WHO classification is the introduction of SMBOTs, which corresponds to the previously called mucinous tumors in their endocervical variant. The genetic‐molecular pattern of SMBOTs, macroscopic appearance, behavior, and precursors have proven to be completely different from MBOTs, which today only refers to the intestinal‐type.^8^

Transvaginal ultrasound (TVUS) is the worldwide first‐line and gold standard imaging technique for the diagnosis of adnexal tumors. It is a reliable, safe, inexpensive, and easily accessible method for the diagnosis of ovarian BOTs, as well as for the subsequent follow‐up of women after surgical treatment.^9^ Several studies are available in literature describing the main sonographic characteristics of BOTs.^10^, ^11^, ^12^, ^13^, ^14^ However, evidence describing the specific ultrasound features of ovarian SMBOTs after the 2014 WHO classification remains limited. In addition, although an association between SMBOTs and endometriosis has been reported,^15^ the relationship with specific types and localization of endometriotic lesions has not been systematically investigated.

Therefore, the aim of this study is: first, to further characterize the clinical and ultrasound features of SMBOTs according to the current WHO classification and compare them with other BOT histotypes; second, to evaluate the association between SMBOTs and different types of endometriotic lesions, with particular attention to their anatomical localization.

## 2 MATERIAL AND METHODS

This is a single‐center, observational, retrospective cohort study, following an a priori study protocol and based on a convenience sample. The whole study was reported according to the Strengthening the Reporting of Observational studies in Epidemiology (STROBE) statement and checklist.^16^

Medical records and electronic clinical databases were screened for all consecutive eligible women with histological diagnosis of BOT who underwent preoperative ultrasound examination by an experienced sonographer^17^ and referred to the Division of Gynecology and Human Reproduction Physiopathology, IRCCS Azienda Ospedaliero‐Universitaria di Bologna, Bologna, Italy, from May 1, 2015 to December 31, 2022 were included.

The inclusion criteria were: age ≥18 years old, absence of pregnancy, availability of good quality gray‐scale and color/power Doppler images and/or videos from preoperative TVUS.

Exclusion criteria wereas follows: histology different from BOTs and the presence of gynecological malignancy.

### 2.1 Clinical data and ultrasound evaluations

Data extracted from preoperative ultrasound, surgical, and histological reports included age at diagnosis, body mass index (BMI), parity, menopausal status, and preoperative serum levels of carbohydrate antigen 125 (CA‐125) and carbohydrate antigen 19.9 (CA‐19.9) (U/mL). Additional factors analyzed were lesion laterality (right, left, or both), FIGO stage, type of surgical treatment (unilateral or bilateral ovarian cystectomy, unilateral or bilateral adnexectomy, and other procedures), presence and localization of endometriosis (peritoneal superficial, deep, or ovarian), and the presence of endometriosis in the BOT‐affected ovary. The histological type of BOT was assessed on the final surgical specimen by experienced gynecological pathologists according to 2014 WHO criteria,^8^ while disease staging was assigned according to the 2014 FIGO staging system for ovarian cancer.^18^

Preoperative transvaginal and transabdominal ultrasound images and/or videos and/or reports were analyzed for all women. All scans were performed using GE Healthcare, Zipf, Austria ultrasound system equipped with a 7.5 MHz transvaginal probe with three‐dimensional facility and a 3.5 to 5 MHz convex probe. Sonographic images and videos were reviewed by two expert ultrasound specialists (S.D.F. and L.D.M.).^17^ In cases of uncertainty or disagreement, a third expert examiner (C.L.) was consulted. All reviewers were blinded to the histopathological diagnoses. All adnexal lesions were described using the International Ovarian Tumor Analysis (IOTA) terms and definitions.^19^ The type of adnexal lesion (unilocular cyst, unilocular‐solid cyst, multilocular cyst, multilocular solid cyst, solid tumor), its maximum diameter, the number of locules, the echogenicity of the cyst fluid [anechoic, low‐level, ground‐glass, mixed, no cyst fluid (solid)], the presence and number of papillary projections and/or solid components, the maximum diameter of the largest solid component, the presence of acoustic shadows and ascites were described. In case of bilateral adnexal lesions or multiple lesions in the same ovary, the largest one was included in the analysis.

Color Doppler ultrasound examination was applied with the same setting in all women: Doppler's pulse repetition frequency was always between 0.3–0.6 KHz; color Doppler gain was set just below the appearance of color Doppler artifacts. A semiquantitative subjective assessment of the amount of blood flow of the solid components/papillary projections was performed, according to IOTA Color Score (CS): CS 1 (no flow), CS 2 (poor flow), CS 3 (moderate flow), and CS 4 (abundant flow).^19^

The subjective assessment of the sonographer about the risk of malignancy of the lesion at first evaluation was documented as certainly benign, probably benign, probably malignant, malignant, uncertain, undescribed.

### 2.2 Study outcomes

The primary study outcome was to analyze the clinical and ultrasound characteristics of SMBOTs after the 2014 WHO histological classification, comparing them with SBOTs and MBOTs. The secondary outcome was to evaluate the association of SMBOTs with different types of endometriotic lesions (ovarian, superficial, and deep).

### 2.3 Statistical analysis

Categorical variables were summarized as counts and percentages, while numerical variables were summarized as medians.

To investigate the presence of differences in baseline characteristics in the different main groups, we performed nonparametric tests, i.e., chi‐squared test or Fisher's exact test for categorical data and Kruskal–Wallis test (comparison of three groups) or Wilcoxon–Mann–Whitney test (comparison of two groups) for numerical data. The variables of greatest interest were also studied as dichotomous variables in the individual modalities to identify statistically significant differences between groups. Two‐tailed *p* values <0.05 were considered statistically significant. For the main clinical and sonographic features, odds ratios (ORs) with 95% confidence intervals (CIs) were calculated to quantify associations between BOT subtypes. Pairwise comparisons were performed between SMBOTs and SBOTs, and between SMBOTs and MBOTs. Analyses were made using IBM SPSS 27.0 (Armonk, NY: IBM Corp).

## 3 RESULTS

### 3.1 Study population

During the study period, 173 women were diagnosed with BOTs. Twenty‐two (13%) women were excluded from the study since they did not meet the selection criteria: 12 (7%) had images of insufficient quality, 8 (5%) had only written ultrasound reports without available images, and 2 (1%) were younger than 18 years. As a result, a total of 151 (87%) women were included in the study. Ninety‐six (63.6%) had SBOTs, 27 (17.9%) MBOTs, 23 (15.2%) SMBOTs, 2 (1.3%) clear cell, 2 (1.3%) Brenner BOTs, and 1 (0.7%) endometrioid. Due to the limited number of cases in the last three histotypes mentioned, we included only the three most common groups (SBOTs, MBOTs, and SMBOTs) for statistical analysis, totaling 146 women.

Table 1 presents the demographic and clinical characteristics of the study population, categorized by different histotypes. The majority of women were premenopausal (62%) with a median age at diagnosis of 44 (32–47) years. Median age at diagnosis in women with SMBOTs was 45 (34–57) years.

**TABLE 1:** Clinical characteristics of 146 women with serous, seromucinous, and mucinous borderline ovarian tumors (BOTs) and comparison between histotypes.

Characteristic	All (*n* = 146)	SBOTs (*n* = 96)	MBOTs (*n* = 27)	SMBOTs (*n* = 23)	SBOTs vs SMBOTs (*p* value)	MBOTs vs SMBOTs (*p* value)	Difference among 3 groups (*p* value)
Median age at diagnosis (years)	44 [32–57]	43 [32–58]	44 [32–58]	45 [34–57]	NS	NS	NS
BMI, kg/m^2^	23 [21–27]	23 [21–27]	23 [21–27]	23 [20–27]	NS	NS	NS
Menopause	55 (38)	38 (40)	10 (37)	7 (30)	0.42	0.77	0.72
CA‐125 >35 U/mL	55 (38)	42 (44)	6 (22)	7 (30)	0.35	0.54	0.09
CA‐19.9 >37 U/mL	22 (15)	5 (5)	7 (26)	10 (43)	<0.001	0.24	0.001
Laterality							
Right	49 (34)	35 (36)	10 (37)	4 (17)	0.09	0.21	0.20
Left	60 (41)	28 (29)	17 (63)	15 (65)	0.003	1	0.001
Bilateral	37 (25)	33 (31)	0 (0)	4 (17)	0.21	0.04	0.002
FIGO stage							
IA	96 (65)	55 (57)	25 (93)	16 (70)			
IB	15 (10)	1 (11)	0 (0)	4 (17)			
IC1	13 (8)	10 (10)	1 (4)	2 (8)			
II	21 (14)	19 (20)	1 (4)	1 (4)			
IIIA1	1 (1)	1 (1)	0 (0)	0 (0)			
Surgical treatment							
Unilateral ovarian cystectomy	23 (16)	16 (17)	6 (22)	1 (4)			
Bilateral ovarian cystectomy	13 (9)	7 (7)	1 (4)	5 (22)			
Unilateral adnexectomy	48 (33)	31 (33)	10 (37)	7 (30)			
Bilateral adnexectomy	59 (40)	39 (40)	10 (37)	10 (43)			
Other types of pelvic surgery^a^	3 (2)	3 (3)	0 (0)	0 (0)			
Surgical treatment in women' with endometriosis ipsilateral to BOT							
Monolateral/Bilateral Ovariectomy/adnexectomy	13 (81)	4 (80)	2 (100)	7 (78)	0.58	0.006	0.02
Monolateral/Bilateral Cystectomy	3 (19)	1 (20)	0 (0)	2 (22)	0.78	0.02	0.04

*Note*: Values are *n* (%) or median [interquartile range].

Abbreviations: BMI, body mass index; CA‐125, carbohydrate antigen 125 and CA 19.9 carbohydrate antigen 19.9; CS, Color Score; IOTA, International Ovarian Tumor Analysis; MBOTs, mucinous ovarian borderline tumors; SBOTs, serous borderline ovarian tumors; SMBOTs, seromucinous borderline ovarian tumors.

^a^ Hysterectomy plus bilateral adnexectomy, bilateral pelvic lymphadenectomy.

### 3.2 Study outcomes

Preoperative serum values of CA‐125 were increased (>35 U/mL) in about one‐third of all women with BOTs (55/146, 38%), with no differences between the three groups. In contrast, serum CA‐19.9 levels (>37 U/mL) were increased in 15% (22/146) of cases, with the highest frequency observed in SMBOTs (43%, 10/23, *p* value <0.001 compared to SBOTs). No significant difference was found between MBOTs and SMBOTs (*p* = 0.24).

Regarding lesion laterality, 41% of BOTs were left‐sided (60/146), 34% right‐sided (49/146), and 25% bilateral (37/146). Notably, SMBOTs were predominantly left‐sided (65%, 15/23), and this laterality difference was statistically significant when compared with SBOTs (*p* = 0.001). Ultrasonographic characteristics of women with serous, seromucinous, and mucinous BOTs and comparison between histotypes are shown in Table 2. According to the IOTA classification, 20% (29/146) of the masses were unilocular, 35% (51/146) unilocular solid, 19% (28/146) multilocular, 23% (34/146) multilocular solid, and 3% (4/146) solid tumors.

**TABLE 2:** Ultrasound findings in 146 women with serous, seromucinous, and mucinous borderline ovarian tumors (BOTs) and comparison between histotypes.

Characteristic	All (*n* = 146)	SBOTs (*n* = 96)	MBOTs (*n* = 27)	SMBOTs (*n* = 23)	SBOTs vs SMBOTs (*p* value)	MBOTs vs SMBOTs (*p* value)	Difference among 3 groups (*p* value)
Type of tumor							
Unilocular	29 (20)	18 (19)	6 (22)	5 (22)	0.77	1	0.90
Unilocular‐solid	51 (35)	41 (43)	3 (11)	7 (30)	0.35	0.15	0.009
Multilocular	28 (19)	12 (13)	13 (48)	3 (13)	1	0.01	<0.001
Multilocular‐solid	34 (23)	22 (23)	5 (19)	7 (30)	0.43	0.51	0.60
Solid	4 (3)	3 (3)	0 (0)	1 (4)	0.58	0.46	0.60
Maximum tumor diameter (mm)	87 [4–600]	80 [4–600]	119 [15–300]	84 [10–330]	0.72	0.02	0.002
Echogenicity of the cyst fluid							
Anechoic	41 (28)	30 (31)	8 (30)	3 (13)	0.12	0.19	0.21
Low level	76 (52)	49 (51)	17 (63)	10 (43)	0.64	0.25	0.37
Ground glass	21 (14)	10 (10)	2 (7)	9 (39)	0.002	0.01	0.001
Mixed	4 (3)	4 (4)	0 (0)	0 (0)	1		0.34
No cyst fluid (solid tumors)	4 (3)	3 (3)	0 (0)	1 (4)	0.58	0.46	0.60
Presence of papillary projections/solid component							
1	49 (34)	32 (33)	6 (22)	11 (48)	0.23	0.08	0.16
2	10 (7)	9 (9)	1 (4)	0 (0)	0.20	1	0.21
3	5 (3)	4 (4)	0 (0)	1 (4)	1	0.46	0.55
	22 (15)	20 (21)	1 (4)	1 (4)	0.07	1	0.03
Largest solid component (mm)	14 [2–250]	24 [2–250]	27.8 [4–99]	23 [8–76]	0.38	0.09	0.007
Vascularization of solid component/papillary projection							
CS1	30 (21)	25 (38)	2 (25)	3 (20)	0.27	0.65	0.67
CS2	26 (17)	21 (32)	2 (25)	3 (20)	0.56	0.65	0.18
CS3	26 (17)	17 (26)	1 (12)	8 (53)	0.09	0.007	0.02
CS4	3 (2)	3 (4)	0 (0)	0 (0)	1	NA	0.45
Information not available	4 (4)	0 (0)	3 (38)	1 (7)			
Number of loci							
2–10	36 (55)	25 (69)	8 (22)	3 (8)	<0.001	0.19	<0.001
>10	26 (22)	9 (35)	10 (38)	7 (27)	1	0.56	0.764
Acoustic shadows	7 (5)	4 (4)	1 (4)	2 (9)	1	1	0.99
Ascites	6 (4)	2 (2)	3 (11)	1 (4)	0.48	0.61	0.11
Diagnosis based on subjective assessment							
Benign	28	13	10	5	1	1	0.92
Probably benign	36	15	14	6	1	0.58	0.53
Probably malignant	35	20	9	6	0.60	0.77	0.48
Malignant	13	6	3	3	0.20	1.00	0.25
Uncertain	11	6	4	2	0.63	0.39	0.11
Not described	23	9	10	4			

*Note*: Values are *n* (%) or median [interquartile range].

Abbreviations: CS, Color Score; IOTA, International Ovarian Tumor Analysis; MBOTs, mucinous ovarian borderline tumors; NA, not applicable; SBOTs, serous borderline ovarian tumors; SMBOTs, seromucinous borderline ovarian tumors.

**FIGURE 1:**
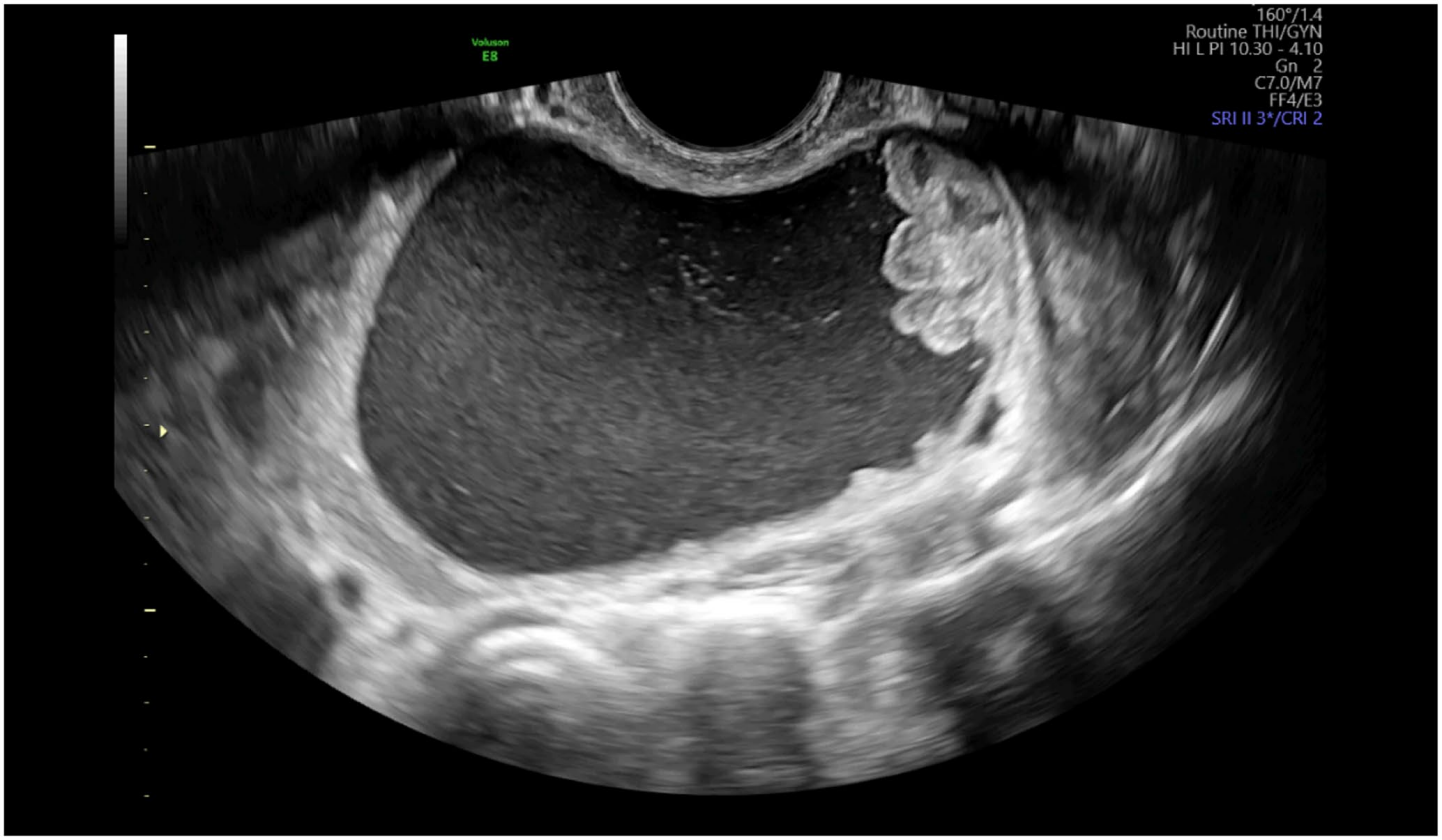
Transvaginal gray scale ultrasound image showing an axial view of a seromucinous borderline ovarian tumor, that appears as an unilocular solid cyst with ground‐glass content and one papillary projection.

**FIGURE 2:**
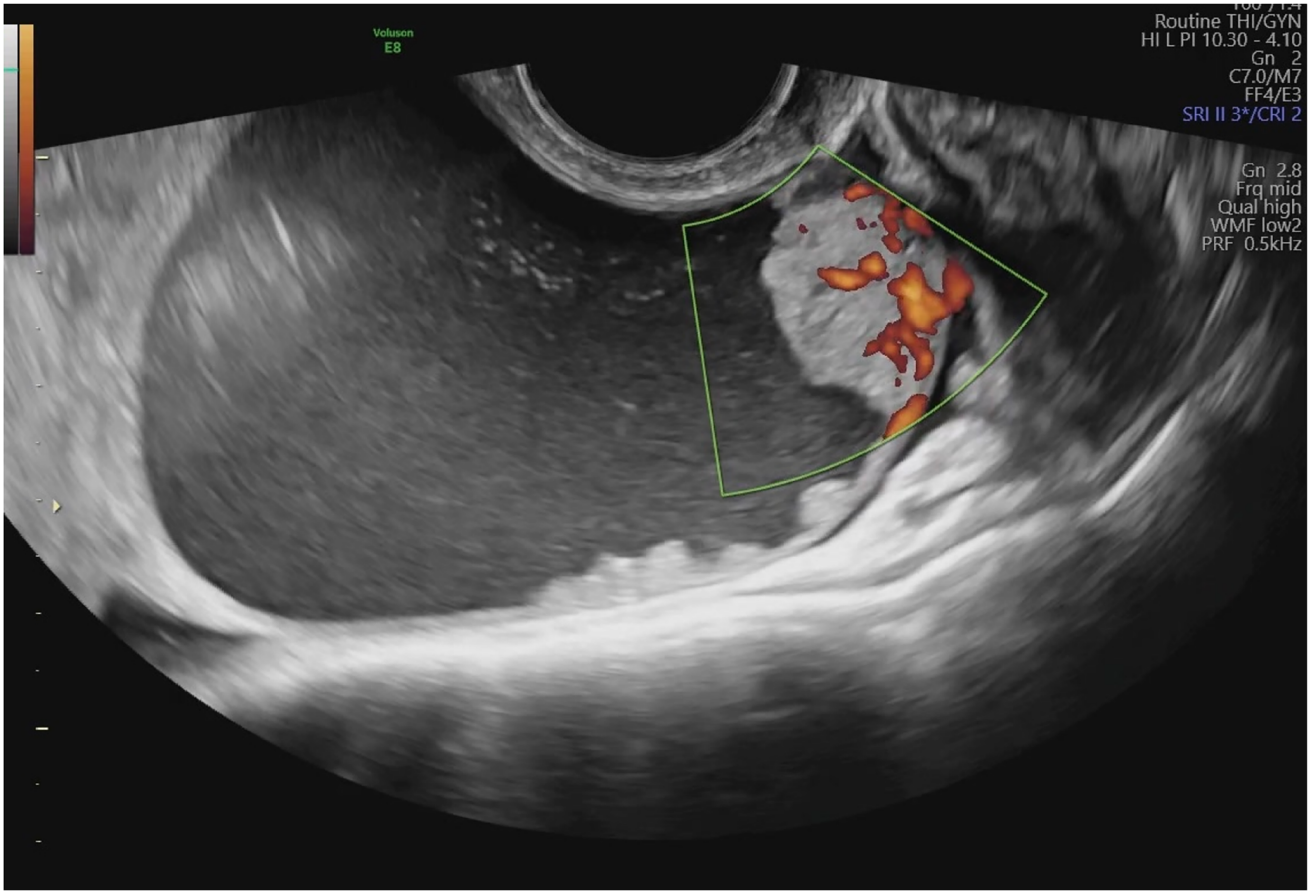
Transvaginal ultrasound image, in axial view, of a seromucinous borderline ovarian tumor, showing a left unilocular solid cyst with ground‐glass content and the presence of one papillary projection with moderate vascularization at Color Doppler (Color Score 3).

**FIGURE 3:**
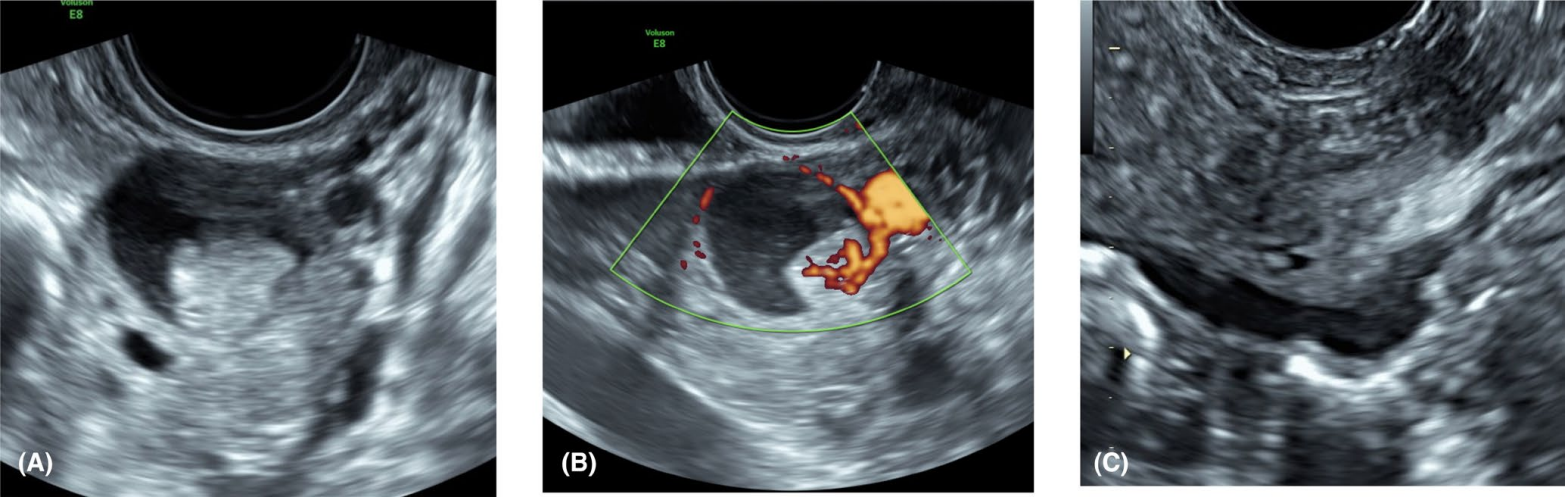
Series of transvaginal ultrasound images from the same woman showing a unilocular solid cyst of the left ovary (gray‐scale modality, image A) containing one papillary projection with moderate vascularization at color Doppler (Color Score 3) (image B). Image C shows deep endometriosis of the posterior compartment involving the anterior rectal wall. Histopathological analysis of the excised left ovarian cyst revealed a seromucinous borderline tumor with involvement of the ovarian serosal surface, arising within an endometriotic cyst.

Comparing the three groups, SBOTs were more frequently unilocular solid masses, while MBOTs were more commonly multilocular masses. SMBOTs were primarily unilocular solid or multilocular solid masses with papillary projections. (Figures 1, 2, 3) The median tumor diameter was significantly larger in MBOTs compared with the other histotypes (*p* = 0.002).

The cyst fluid echogenicity was most commonly low‐level (76/146, 52%), whereas ground‐glass content was significantly more frequent in SMBOTs (9/23, 39%, *p* = 0.001) (Figure 1).

Regarding the vascularization of solid components/papillary projections, SMBOTs exhibited moderate vascularization (CS3) (Figure 2) in 53% of cases, which was significantly higher than SBOTs (26%) and MBOTs (12%) (*p* = 0.02).

When effect sizes were quantified (Table S2), SMBOTs were significantly more likely than SBOTs to show ground‐glass echogenicity (OR 11.5, 95% CI 3.6–37.1), CS3 vascularization of papillations/solid components (OR 5.2, 95% CI 2.0–13.7). Similar associations were observed when comparing SMBOTs with MBOTs for CS3 vascularization of papillations/solid components (OR 4.4, 95% CI 1.3–14.8). A complete overview of all calculated ORs and CIs is provided in Table S2.

### 3.3 Association with endometriosis

**FIGURE 4:**
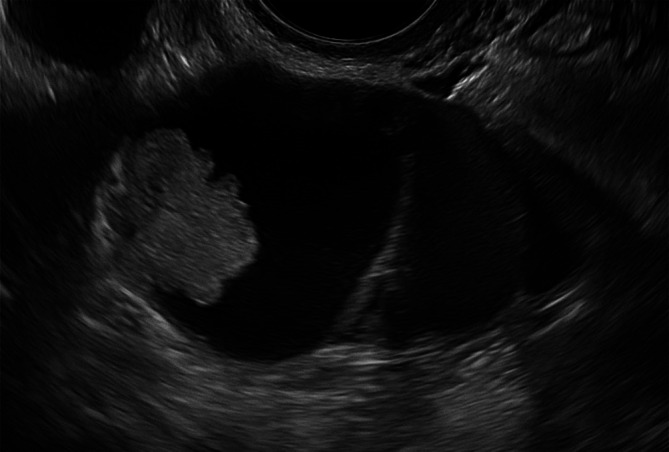
Transvaginal gray‐scale ultrasound image, in axial view, of a seromucinous borderline ovarian tumor (lateral) with adjacent endometrioma (medial) of the right ovary.

Data regarding the association of BOTs with endometriosis are shown in Table 3. Endometriosis was present in 25% (37/146) of all BOTs with a significantly higher frequency in SMBOTs (70%, 16/23, Figures 3 and 4) compared to SBOTs (17%, 16/96; *p* < 0.001) and MBOTs (19%, 5/27; *p* < 0.001). No significant differences in the type of endometriotic lesions were observed among the groups.

**TABLE 3:** Association with endometriosis and type of endometriosis in 146 patients with serous, seromucinous, and mucinous borderline ovarian tumors (BOTs).

	All (*n* = 146)	SBOTs (*n* = 96)	MBOTs (*n* = 27)	SMBOTs (*n* = 23)	SBOTs vs SMBOTs (*p* value)	MBOTs vs SMBOTs (*p* value)	Difference among 3 groups (*p* value)
Presence of endometriosis	37 (25)	16 (17)	5 (19)	16 (70)	<0.001	<0.001	<0.001
Type of endometriosis^a^							
Ovarian endometriosis only	19 (51)	7 (44)	2 (40)	10 (62)	0.48	0.61	0.49
Deep endometriosis only	4 (11)	2 (12)	1 (20)	1 (6)	1	0.43	0.66
Superficial and ovarian endometriosis	7 (19)	3 (19)	2 (40)	2 (12)	1	0.23	0.39
Superficial endometriosis only	7 (19)	4 (25)	0 (0)	3 (19)	1	0.55	0.46

*Note*: Values are *n* (%) or median [interquartile range].

Abbreviations: MBOTs, mucinous ovarian borderline tumors; SBOTs, serous borderline ovarian tumors; SMBOTs, seromucinous borderline ovarian tumors.

^a^ No cases of deep endometriosis plus ovarian endometriosis were found in the three groups.

Presence and localization of ovarian endometriosis in women with concomitant ovarian endometriosis and BOT is shown in Table 4. Regarding the concomitant presence of endometriosis and BOT in the same ovary (16 women), the presence of ovarian endometriosis in the ovarian parenchyma outside BOT's cyst (Ovarian endometriosis OUTside of BOT; 15/16) was diagnosed with TVUS in cases of ovarian endometriomas (3/15), while in the majority of cases (12/15) it was identified histologically only. A significant difference was observed between SBOTs (100% 5/5) and SMBOTs (89%, 8/9, *p* = 0.04) when analyzing ovarian endometriosis located outside the ipsilateral BOT. In the patient who had ovarian endometriosis inside the ipsilateral BOT (Ovarian endometriosis INside BOT; 1/16), the ultrasound suspected the presence of BOT and associated endometriosis, and the diagnosis was confirmed at histology. No significant difference between the tumor histotypes was found (*p* = 0.66).

**TABLE 4:** Presence and localization of ovarian endometriosis in 26 women with concomitant ovarian endometriosis and BOT.

	All (*n* = 26)	SBOTs (*n* = 10)	MBOTs (*n* = 4)	SMBOTs (*n* = 12)	SBOTs vs SMBOTs (*p* value)	MBOTs vs SMBOTs (*p* value)	Difference among 3 groups (*p* value)
Presence of endometriosis in BOT's ipsilateral ovary	16 (62)	5 (50)	2 (50)	9 (75)	0.42	0.58	0.60
Ovarian endometriosis INside ipsilateral BOT	1/16 (6)	0/5 (0)	0/2 (0)	1/9 (11)	1	1	0.66
Ovarian endometriosis OUTside of ipsilateral BOT	15/16 (94)	5/5 (100)	2/2 (100)	8/9 (89)	0.04	0.21	0.03
Presence of endometriosis in BOT's contralateral ovary	10 (38)	3/10 (30)	3/10 (30)	4/10 (40)	1	1	0.9

Abbreviations: MBOTs, mucinous ovarian borderline tumors; SBOTs, serous borderline ovarian tumors; SMBOTs, seromucinous borderline ovarian tumors.

Lastly, in women who had endometriosis in BOT's contralateral ovary (10 women), ovarian endometriomas (7/10) were diagnosed at TVUS and confirmed histologically, while the presence of superficial ovarian endometriosis or endometriosis in the ovarian parenchyma (3/10) was diagnosed at histology only. No significant difference between the tumor histotypes was found (*p* = 0.9).

When effect sizes were quantified (Table S2) with regard to endometriosis, SMBOTs were strongly associated with its presence compared to both SBOTs (OR 6.5, 95% CI 2.4–17.4) and MBOTs (OR 5.7, 95% CI 1.6–20.5). The association was even stronger when considering endometriosis located outside the BOT cyst within the same ovary (SBOTs vs. SMBOTs: OR 6.8, 95% CI 2.1–21.5; MBOTs vs. SMBOTs: OR 6.7, 95% CI 1.3–35.7).

## 4 DISCUSSION

This study identifies specific clinical and ultrasonographic features associated with SMBOTs and provides additional evidence supporting their distinction from other BOT histotypes. The novelty of our study lies in the evaluation of SMBOTs according to the 2014 WHO classification, integrating sonographic findings with clinical and pathological data, including the detailed assessment of their association with endometriosis and its localization.

From a clinical perspective, our findings suggest that the combination of ultrasound morphology, vascularization patterns, serum biomarkers, and the presence of endometriosis may improve preoperative identification of SMBOTs and contribute to a more tailored surgical approach.

Compared to other BOT histotypes, SMBOTs were more frequently left‐sided, unilateral, and exhibited more frequently elevated serum CA‐19.9 levels. At ultrasound, they more often appeared as unilocular‐solid or multilocular‐solid ovarian cysts with ground‐glass content (Figure 1) and moderate vascularization (CS3) of papillary projections or solid components (Figure 2). Pathologically, a notable association with endometriosis was observed (Figure 3), particularly involving the ipsilateral ovarian parenchyma outside the BOT cyst (Figure 4).

In contrast to previous studies reporting SMBOTs as more commonly bilateral adnexal lesions,^20^, ^21^ our findings indicate that they are more frequently present as left‐sided unilateral masses. This left‐sided distribution may be explained by the well‐established association between SMBOTs and endometriosis, which itself more commonly affects the left ovary. Such asymmetry is likely related to anatomical factors, including the presence of the sigmoid colon on the left side, which may impair peritoneal fluid clearance and facilitate the implantation of regurgitated endometrial cells.^22^

The high prevalence of endometriosis observed in our cohort is consistent with prior reports suggesting an association ranging from 30% to 70%.^15^ In our series, endometriotic lesions were most frequently located in the same ovary as the SMBOT, often within the ovarian parenchyma outside the tumor cyst. This spatial relationship may reflect a shared local microenvironment since from a pathophysiological perspective, several clinical and experimental studies support a role for endometriosis as a precursor condition for specific ovarian tumor histotypes. Chronic inflammation, oxidative stress, and alterations of the ovarian microenvironment have been proposed as key mechanisms promoting neoplastic transformation.^20^, ^23^ In addition, molecular data have demonstrated shared alterations in genes such as ARID1A, PTEN, and KRAS,^24^ as well as dysregulation of microRNA families including miR‐200, suggesting common pathogenic pathways between endometriosis and endometriosis‐associated tumors.^25^ However, given the observational and retrospective nature of our study, this remains a hypothesis, and a direct causal relationship cannot be definitively proven. Therefore, further prospective and translational studies are needed to confirm this association and better elucidate the underlying mechanisms.

The preoperative elevation of serum CA‐19.9 in SMBOTs was also a significant finding, in agreement with previous studies that observed similar elevations of CA‐19.9 in SMBOTs and MBOTs, with the latter being well‐documented in the literature for their association with increased levels of this marker.^21^, ^26^

Regarding ultrasound characteristics, a peculiar feature of SMBOTs was the ground‐glass content within the cyst, which was more frequently observed in this histotype compared with both SBOTs and MBOTs. While the cystic content of SMBOTs is usually mucinous at macroscopy, hemorrhagic material may occasionally be present.^15^ On ultrasound, this finding may mimic the typical appearance of endometriomas.^27^ When comparing the ultrasonographic features of SMBOTs with MBOTs, we found that SMBOTs were generally smaller in size and more likely to present as unilocular or multilocular solid masses, as previously reported.^10^, ^12^, ^21^ On the other hand, SMBOTs and SBOTs presented similarly in terms of size, but SMBOTs more frequently exhibited fewer but more vascularized papillary projections/solid component compared to SBOTs. In particular a significant prevalence of moderate vascularization of papillary projections/solid component (CS 3) was found in SMBOTs when compared to other histotypes. These findings are consistent with other studies, including that by Kurata et al.,^28^ which found that SBOTs tend to have more papillary projections on MRI, and Pascual et al.,^29^ who described clinical and ultrasound features of mucinous ovarian tumors, finding that borderline tumors (including the seromucinous histotype, previously called endocervical‐like mucinous BOT) exhibited CS3 or 4 more frequently than benign tumors. Nonetheless, the distinction between SMBOTs and SBOTs remains challenging, as they share several ultrasonographic features.^30^ Our findings emphasize that a more accurate differential diagnosis can be achieved by integrating sonographic assessment with clinical characteristics (i.e., laterality of the lesion), biochemical markers (i.e., CA‐19.9), and pathological associations with endometriosis.

From an implementation standpoint, these findings may have direct implications for clinical practice. In the presence of an adnexal mass showing ground‐glass echogenicity, papillary projections with moderate vascularization, and elevated CA‐19.9 levels, particularly in association with known or suspected endometriosis, the possibility of SMBOT should be considered.

This integrated assessment may support more accurate preoperative risk stratification, guide referral to specialized centers, and assist in surgical planning, especially when fertility preservation or concomitant treatment of endometriosis is required.

To the best of our knowledge, this is the first study to investigate the clinical and ultrasound characteristics of histologically confirmed SMBOTs since the 2014 WHO histological classification. The integration of standardized ultrasound assessment with clinical and histopathological data represents a strength of the study. However, several limitations should be acknowledged. The retrospective single‐center design may have introduced selection bias, as cases referred to a tertiary center are often more complex and may not reflect the broader population of women with BOTs. In particular, as our institution is a referral center for endometriosis, referral bias may have contributed to the high prevalence of endometriosis observed in the SMBOT group. Information bias should also be considered, as ultrasound evaluation was based mainly on stored images rather than real‐time examinations, potentially leading to misclassification of some morphological features (e.g., small papillary projections within large cysts). Nevertheless, the use of standardized IOTA terminology and the blinded review by expert sonographers likely improved the reliability of the assessments.

In addition, most women with histologically confirmed endometriosis and BOT in the same ovary underwent adnexectomy or oophorectomy. Therefore, in cases treated with cystectomy alone, endometriotic foci in the residual ovarian tissue may have been underdetected.

The exclusion of less frequent BOT histotypes, although necessary due to small sample size, may limit the generalizability of the findings. Furthermore, no a priori sample size calculation was performed; therefore, the study should be considered exploratory and hypothesis‐generating, and the findings will need confirmation in larger, prospective cohorts.

Potential confounding factors should also be considered. Patient age and menopausal status may have influenced both tumor distribution and sonographic appearance. Moreover, some ultrasound features identified in SMBOTs, such as ground‐glass echogenicity or lesion laterality, may be partly related to the coexistence of endometriosis rather than to the tumor histotype itself.

These findings have potential clinical implications for the preoperative assessment of adnexal masses. The identification of specific ultrasound patterns, in combination with clinical and biochemical data, may support the suspicion of SMBOT and assist in surgical planning.

Further studies are required to clarify whether the association between SMBOTs and endometriosis reflects a causal relationship or a frequent coexistence. In particular, prospective and translational investigations integrating clinical, imaging, and molecular data may help to better define the underlying pathogenetic mechanisms.

In parallel, advances in imaging analysis, including the application of machine learning models to ultrasound and MRI, may improve diagnostic accuracy and reduce interobserver variability, particularly in the evaluation of vascularization and solid components.

## 5 CONCLUSION

In conclusion, SMBOTs appeared more frequently as unilateral, left‐sided, unilocular‐solid or multilocular‐solid cysts with ground‐glass content, more commonly containing a single papillary projection or solid component with moderate vascularization. Elevated preoperative serum CA‐19.9 levels and the strong association with endometriosis, particularly when both occur in the same ovary, provide important diagnostic clues for clinicians.

These findings suggest that the integration of ultrasound features with clinical and biochemical data may improve the preoperative identification of SMBOTs. In particular, the recognition of specific sonographic patterns in association with endometriosis should prompt a comprehensive evaluation aimed at detecting concomitant endometriotic lesions.

This approach may support individualized patient counseling and optimize surgical management by addressing both the borderline tumor and associated endometriosis during the same procedure.

## AUTHOR CONTRIBUTIONS

Simona Del Forno and Renato Seracchioli conceived and designed the study. Marisol Doglioli, Giovanna Palomba, Claudia Vicenzi, Matilde Antonelli, Francesca Govoni, and Roberto Paradisi contributed to data acquisition. Simona Del Forno, Lucia De Meis, and Chiara Landolfo analyzed and interpreted the ultrasound data. Marisol Doglioli, Giovanna Palomba, and Simona Del Forno drafted the article. Diego Raimondo, Chiara Landolfo, and Renato Seracchioli critically revised the manuscript for important intellectual content. All authors gave final approval of the version to be published and agreed to be accountable for all aspects of the work.

## FUNDING INFORMATION

The authors declare no funding.

## CONFLICT OF INTEREST STATEMENT

The authors declare no conflict of interest.

## ETHICS STATEMENT

The study was approved by the local Ethics Committee (CE AVEC; reference number: 443/2023/Oss/AOUBo) on August 8, 2023. The research was carried out in accordance with the Helsinki Declaration, and all women signed an informed consent for the use of their data for clinical research.

## Supporting information


**Figure S1.** Flow Diagram of patient selection.

**Table S2.** Associations between BOT subtypes and selected clinical and sonographic features.

## Data Availability

The data underlying this article are available in 10.5281/zenodo.18405450.
