# Supplementary material for: Seromucinous borderline ovarian tumors: Clinical and ultrasound characteristics and association with endometriosis
Source: Acta Obstet Gynecol Scand. 2026 May 26;105(9):1860–70. doi: 10.1111/aogs.70254 (PMC13395054; doi:10.1111/aogs.70254)
Supplement: Supplementary file 1 [file AOGS-105-1860-s002.docx]

**Supplementary Table 2.** Associations between BOT subtypes and selected clinical and sonographic features

| **Feature** | **SMBOT vs SBOT** | **SMBOT vs MBOT** |
| --- | --- | --- |
| **CA-19.9 >37 U/ml** | 3.04 (0.97–9.47) | 1.24 (0.34–4.53) |
| **Left-sided tumors** | 4.55 (1.74–11.94) | 1.74 (0.56–5.46) |
| **Unilocular-solid tumors** | 4.23 (1.38–12.99) | 1.25 (0.36–4.31) |
| **Ground-glass echogenicity** | 11.54 (3.59–37.08) | 3.38 (0.95–12.10) |
| **Vascularization CS3** | 5.23 (2.00–13.71) | 4.44 (1.34–14.77) |
| **Endometriosis associated to SMBOTs** | 6.50 (2.43–17.38) | 5.72 (1.60–20.45) |
| **Endometriosis OUTside SMBOT cysts** | 6.78 (2.14–21.47) | 6.67 (1.25–35.65) |

*Abbreviations:* SBOTs, serous borderline ovarian tumors; MBOTs, mucinous ovarian borderline tumors; SMBOTs, seromucinous borderline ovarian tumors, CI, confidence intervals

*Notes:* Values are expressed in OR (95% CI*)*
