# Supplementary figures and images for: Seromucinous borderline ovarian tumors: Clinical and ultrasound characteristics and association with endometriosis
Source: Acta Obstet Gynecol Scand. 2026 May 26:10.1111/aogs.70254. Online ahead of print. doi: 10.1111/aogs.70254 (PMC13395054; doi:10.1111/aogs.70254)

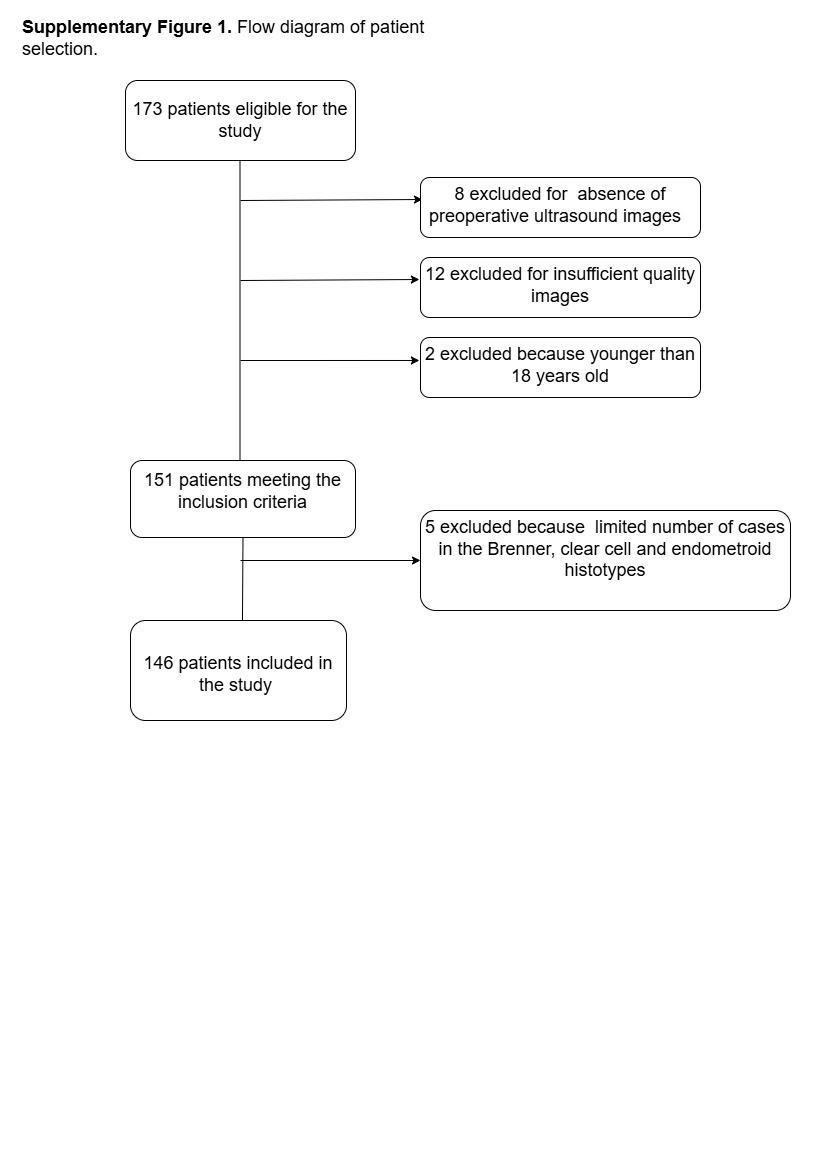

Supplement: Supplementary file 1 — Figure S1. Flow Diagram of patient selection. [file AOGS-9999-0-s002.jpg]
